# Capsid Assembly Modulators as Antiviral Agents against HBV: Molecular Mechanisms and Clinical Perspectives

**DOI:** 10.3390/jcm11051349

**Published:** 2022-03-01

**Authors:** Valerio Taverniti, Gaëtan Ligat, Yannick Debing, Dieudonne Buh Kum, Thomas F. Baumert, Eloi R. Verrier

**Affiliations:** 1 Université de Strasbourg, Inserm, Institut de Recherche sur les Maladies Virales et Hépatiques UMR_S1110, 67000 Strasbourg, France; vtaverniti@unistra.fr (V.T.); gligat@unistra.fr (G.L.); thomas.baumert@unistra.fr (T.F.B.); 2 Aligos Belgium BV, 3001 Leuven, Belgium; ydebing@aligos.com (Y.D.); dkum@aligos.com (D.B.K.); 3 Institut Hospitalo-Universitaire, Pôle Hépato-Digestif, Nouvel Hôpital Civil, 67000 Strasbourg, France

**Keywords:** core protein, core protein allosteric modulators, drug discovery and development, therapeutics

## Abstract

Despite a preventive vaccine being available, more than 250 million people suffer from chronic hepatitis B virus (HBV) infection, a major cause of liver disease and HCC. HBV infects human hepatocytes where it establishes its genome, the cccDNA with chromosomal features. Therapies controlling HBV replication exist; however, they are not sufficient to eradicate HBV cccDNA, the main cause for HBV persistence in patients. Core protein is the building block of HBV nucleocapsid. This viral protein modulates almost every step of the HBV life cycle; hence, it represents an attractive target for the development of new antiviral therapies. Capsid assembly modulators (CAM) bind to core dimers and perturb the proper nucleocapsid assembly. The potent antiviral activity of CAM has been demonstrated in cell-based and in vivo models. Moreover, several CAMs have entered clinical development. The aim of this review is to summarize the mechanism of action (MoA) and the advancements in the clinical development of CAMs and in the characterization of their mod of action.

## 1. Introduction

Although a preventive vaccine exists against hepatitis B virus (HBV), around 250 million of people around the world suffer chronic HBV infection, the principal cause of advanced liver disease and hepatocellular carcinoma (HCC) [1,2].

The approved antiviral therapies, Nucleos(t)ide analogues (NUCs), including lamivudine, adefovir, tenofovir, or entecavir, can effectively control viral replication by directly inhibiting the reverse transcriptase activity of the HBV polymerase. However, the treatment is lifelong, and a viral cure remains extremely rare. Pegylated interferon-α (PEG-IFN-α)-based therapies can result in a viral cure in a small number of patients but they have significant side effects [3].

HBV is a small DNA virus and belongs to the Hepadnaviridae family. The genome of HBV is a partially double-stranded circular DNA (3.2 kilobase (kb) pairs) named relaxed circular (rc) DNA. The viral polymerase is covalently attached to the 5′ end of the minus strand of the rcDNA. The viral genome encodes four overlapping open reading frames (ORFs: P, C, S, and X). The P ORF encodes the polymerase (a large protein of 800 amino acids). Depending on whether translation is initiated from the precore region of the preC RNA or the core regions of pregenomic (pg)RNA, the C ORF encodes the hepatitis B e antigen (HBeAg) and the viral nucleocapsid hepatitis B core antigen (HBcAg), respectively. The S ORF encodes the viral surface envelope proteins (HBsAg) and the X ORF encodes the hepatitis B x antigen (HBxAg) [4].

HBV infection involves the attachment and entry of HBV virions into host hepatocytes through heparan sulfate proteoglycan (HSPG), including glypican 5 (GPC5) [5], and the liver-specific sodium-taurocholate cotransporting protein (NTCP) [6,7]. After entry, the HBV nucleocapsid is released into the cytoplasm and transported to the nucleus where it interacts with the nucleoporin complex. The rcDNA is then released into the nucleus where it is converted into an episomal covalently-closed-circular (ccc) DNA minichromosome which serves as a template for HBV RNA transcription [4]. The HBV pgRNA is then incorporated in the newly formed nucleocapsids where it is retrotranscribed in the corresponding rcDNA. The rcDNA-containing nucleocapsid is either enveloped and released as new virions or redirected into the nucleus to replenish the cccDNA pool [4]. Therefore, CHB is characterized by the persistence of a nuclear cccDNA, which is not targeted by approved antiviral agents [8].

Novel therapeutic strategies for curative approaches (inactivation or the loss of HBV cccDNA) are urgently needed. In this context, HBcAg represents a target of choice for the development of new antivirals as it plays a pivotal role in the HBV life cycle. The core protein allosteric modulators (CAM) are currently under clinical development. Nevertheless, their mechanism of action (MoA) remains poorly characterized.

In this review, we summarize the molecular and cellular functions of HBcAg in the key steps of the HBV life cycle and highlight the CAM molecules currently in preclinical/clinical development for novel therapeutic strategies.

## 2. HBV Core Protein: A Multifunctional Protein Essential for HBV Life Cycle

### 2.1. Core Protein Is the Building Block of HBV Nucleocapsid

**Figure 1:**
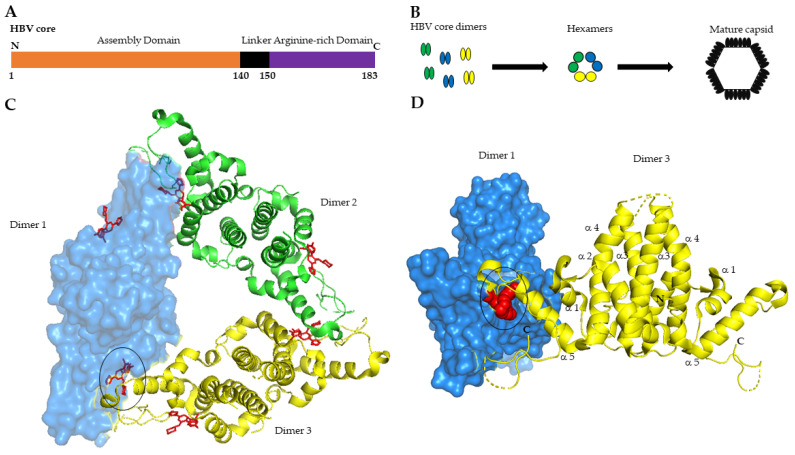
HBV core protein N-terminal domains 3D structure and capsid assembly process. (**A**) Schematic representation of HBV core domains N-terminal: N. C-terminal: C; (**B**) Steps of capsid assembly process; (**C**) Cristal structure of core protein hexamer with six bound NVR-010–001-E2 molecules shown in red. The structure has been adapted from the deposited 3D structure of HBV core mutant Y132A on PDB database (PDB: 5e0i; Lukacs, C.M., Abendroth, J., Klumpp, K., Crystal structure of the HBV capsid Y132A mutant (VCID 8772) in complex with NVR10-001E2 at 1.95A resolution, doi: 10.2210/pdb5e0i/pdb) [19]. Core dimers are colored respectively in blue (dimer 1), green (dimer 2), yellow (dimer 3). Dimer 1 is shown in surface mode. (**D**) Front view of dimer 1 and dimer 3 complex. Dimer 1 is shown as surface mode. (**C**,**D**) CAM molecule NVR-010–001-E2 bound at the dimer 1 and dimer 3 interface is encircled in black. The structure was designed using PyMOL, Version 2.5.2, Schrödinger, LLC.

HBV core protein or HBV core antigen (HBcAg) is a 183-amino acid protein translated from pgRNA. It includes three distinct domains: the alpha-helical rich N-terminal domain (aa 1–140) involved in nucleocapsid assembly, a linker region (aa 141–149), and the arginine-rich C-terminal domain (aa 150–183) required for viral genome replication, although dispensable for capsid formation (Figure 1A) [9].

From a structural point of view, HBcAg constitutes the building block of the nucleocapsid structure. Soon after its translation, the Core assembles into homodimers, while three homodimers interact to form a trimer of dimers (Figure 1B) [10,11]. Structural studies uncovered the architecture of the nucleocapsid shell. The core N-terminal assembly domain comprises five α-helices. Dimerization of core protein is directed by hydrophobic boundaries between α-helices 3 and 4 of two distinct monomers that form a four-helix bundle at the interaction surface (Figure 1D). A cysteine at position 61 creates disulfide bridges in the dimer interface, however, mutational studies have shown that this residue is not essential for dimer-capsid formation [12]. On the other hand, the distal part of the N-terminal assembly domain (α-helix 5) is involved in the interdimer binding (Figure 1C,D). The mature capsid shell is formed by 240-core subunits, the canonical T = 4 structure [12,13,14]. In vitro studies showed that capsid assembly is a fine-tuned allosteric process whose kinetic strictly depends on core dimers concentration, ionic stringency, and temperature (reviewed in [15]). The initial step of capsid assembly is a slow process that ensures the correct interaction of core dimers (nucleation step) as well as the specific binding and encapsidation of the pol-pgRNA complex [16]. Once this complex is established, the fully mature nucleocapsid is built-up and the pgRNA can be reverse transcribed in rcDNA. Perturbations of normal nucleocapsid assembly strongly affect the pgRNA-pol encapsidation [17]. Recently, Luo and co-workers reported that specific mutations in the core α-helix 5, involved in interdimer interaction, affect both capsid assembly as well as pgRNA-pol encapsidation [18]. In this study the authors conclude that pgRNA-pol complex interacts with a capsid intermediate and not with a single core dimer.

### 2.2. Core Is Essential for the HBV Life Cycle

**Figure 2:**
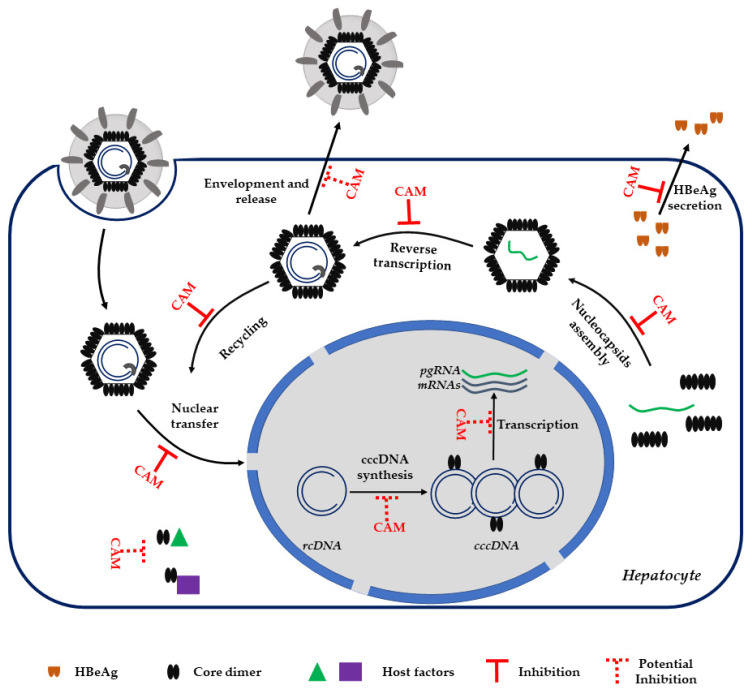
HBV life cycle. The steps regulated by HBV core protein-(HBcAg) and inhibited or potentially inhibited by capsid assembly modulators (CAMs) are indicated. rcDNA: relaxed circular DNA. cccDNA: covalently closed circular DNA. HBeAg: HBV e antigen. pgRNA: pregenomic RNA.

Beyond its molecular function described above, HBcAg protein modulates others important steps in the viral life cycle (Figure 2).

After HBV infection, the nucleocapsid is released into the cytoplasm and translocates to the nucleus where it disassembles and releases the rcDNA-pol complex [20,21]. Nucleocapsid trafficking in the cytoplasm is driven by the core protein through its binding to importin α and β [21,22]. Several studies propose that the C-terminal domain of core, containing a nuclear localization signal (NLS), can be exposed externally of the capsid shell in a phosphorylaton-dependent manner and thus can be recognized by NLS-binding proteins [23,24,25].

It has been recently reported that after HBV infection and nucleocapsid disassembly, the resulting free core protein stably binds to newly synthesized cccDNA, potentially contributing to cccDNA synthesis or stability [26]. Core binds to cccDNA at the level of CpG island 2 [27]. Recruitment of Core to cccDNA induces epigenetic modifications such as hypomethylation of the CpG island 2 that positively correlates with cccDNA transcription and thus viral replication [27,28,29].

The core C-terminal domain (CTD), although dispensable for nucleocapsid assembly, is essential for viral replication [9,30]. The CTD contains clusters of arginine residues conferring a net positive charge; it is mainly localized on the interior of the nucleocapsid and it is able to bind nucleic acids [31]. For these reasons, the CTD is considered having nucleic acid chaperone activity. Indeed, in vitro experiments demonstrated the ability of the core CTD to support the annealing and unwinding of DNA [32]. Moreover, Core is known to be phosphorylated at serine residues present in the CTD and that the phosphorylation status influences its chaperone activity, pgRNA encapsidation, as well as other functions, such as capsid stability and core cellular trafficking [33].

### 2.3. Core Interacts with Host Factors for Its Functions

As described above, HBcAg regulates several steps of the HBV life cycle (Figure 2). To perform such broad and sophisticated functions, Core exploits host-protein properties.

Core dimers interact with human heat shock protein 90 (HSP90). The binding with this chaperone protein facilitates the formation and stabilization of HBV nucleocapsid, thus, HSP90 is considered as a proviral host factor [34]. The NXF1/p15 machinery, generally involved in host RNA nuclear export, directly binds to HBcAg, and drives its export towards the cytoplasm [35].

The CTD phosphorylation regulates Core functions at different levels. Several host kinases have been identified to be involved in Core phosphorylation, notably phosphokinase C (PKC), serine arginine protein kinase 1 (SRPK1), cyclin-dependent kinase 2 (CDK2), SRPKs, Glyceraldehyde-3-phosphate dehydrogenase protein kinase (GAPD-protein kinase), and PLK1 (reviewed in [33]). On the other hand, Hu and coworkers recently identified protein phosphatase 1 (PP1) as being responsible of Core dephosphorylation with a putative implication for pgRNA encapsidation. In this study, the authors found that HBcAg is present in pgRNA-containing nucleocapsids which is hypophosphorylated, whereas HBcAg in empty capsids is hyperphosphorylated [36].

A recent proteomic analysis generated a list of 60 potential partners of Core with the majority being RNA binding protein family. In particular, SRSF10, an RNA binding protein involved in host RNA splicing, directly interacts with Core, and is considered an HBV restriction factor controlling HBV RNA levels [37].

Other host factors have been reported to interact with Core, however, most of them exhibit antiviral functions. The chaperon protein HSP40 binds to Core and induces its degradation, thereby affecting HBV replication [38]. Moreover, Core is targeted by the E3 ubiquitin ligase NIRF that controls its amount through the ubiquitin–proteasome pathway [39]. Lucifora and coworkers described the interaction between HBcAg and the deaminases APOBEC3A and APOBEC3B. These two deaminases are targeted to cccDNA by Core and induce the formation of apurinic sites on the HBV minichromosome, followed by its degradation [40].

## 3. Capsid Assembly Modulators

The lack of efficient, anti-HBV therapies prompted the scientific community to investigate alternative targets broadly and intensively for the development of new direct antiviral agents (DAA).

Due to the central role of HBcAg in the HBV life cycle, particular attention has been paid to the discovery and improvement of capsid assembly modulators (CAMs). Generally, nucleocapsid assembly is characterized by a slow nucleation rate driven by a weak dimer–dimer association. Altering the binding strength and rate of core dimers impacts the whole nucleocapsid assembly. As indicated by the name, the main MoA of CAMs is to impair nucleocapsid assembly by affecting the kinetics and/or the correct interactions between core dimers.

### 3.1. CAM Chemotypes

CAM molecules have been classified into two main families (I and II) depending on the ability to impair nucleocapsid assembly, whereas each family encompasses several different chemotypes. Class I includes CAM chemotypes that induce the misassembly of core dimers giving rise to core aggregates or aberrant capsid structures that do not incorporate a pgRNA-polymerase complex [19,41]. These structures can be identified by different methods, notably electron microscopy and gel filtration [42]. The first and better characterized CAMI compounds were heteroaryldihydropyrimidine (HAP) and its derivatives [43]. However, more recently, a non-HAP CAMI molecule has been described [44]. On the other hand, CAMII molecules are structurally more diverse and comprise phenyl propenamide derivatives (PPA) [45] sulfamoylbenzamides (BAs and SBAs) [46], sulfamoylpyrroloamides (SPAs), and glyoxamoylpyrroloxamides (GLPs) [41]. CAMII molecules stabilize dimer–dimer interaction with the consequent increase in speed of the nucleocapsid assembly kinetic [41,47]. The increasing nucleation speed promotes the formation of normal nucleocapsid but is devoid of the pgRNA-polymerase complex.

Structural studies show that all CAM molecules bind to a hydrophobic pocket located at the core dimerization interface near the C-termini of the core assembly subunits (Figure 1) [19,47]. Filling this pocket by CAMs causes structural changes in the core dimer structure that severely impact the correct capsid assembly and, thus block the internalization of the pgRNA-polymerase and reverse transcription [19].

### 3.2. CAM MoA

As a consequence of core dimer functions, CAM treatment strongly affects all steps of the HBV life cycle (Figure 2). The primary MoA of CAM is to drive the nucleocapsid misassembly. Depending on the CAM chemotype used during treatment, the result is the formation of core aggregates, aberrant, or normal nucleocapsids devoid of the pgRNA-polymerase complex. Thus, CAM compounds directly abrogate viral replication and post-infection spread. Indeed, all studies involving CAM molecules report a strong reduction of both intracellular and extracellular HBV DNA. The efficacy of CAM compounds as DAAs has been demonstrated in vitro as well as in vivo in humanized mice [43,48,49]. Moreover, these molecules actively inhibit the viral replication of NUC-resistant HBV mutants [33]. Interestingly, long-term treatment or treatment with higher concentrations of CAMs induce a strong reduction of viral RNA transcription, HBsAg, and HBeAg secretion, and total cccDNA in both infected HepaRG cells and primary human hepatocytes (PHH) [50,51,52]. Moreover, in contrast to NUC treatment, CAM compounds lead to a reduction of secreted RNA-filled HBV like particles. This class of HBV-like particles is expected to play an immunomodulation function [53,54,55], making their reduction of interest when combined with treatment aiming at restoring an immunological response against HBV.

The second MoA of CAM compounds is to inhibit *de novo* infection. Specifically, time of addition studies in PHH show that the CAMs added together with the viral inoculum strongly reduced cccDNA synthesis with the concomitant reduction of all HBV viral parameters [52,56].

Structural studies reported that CAMs destabilize already assembled nucleocapsid by fulfilling the CAM binding pockets [57]. Moreover, in vitro data showed that the addition of specific HAPs or SBAs can either stabilize or destabilizes the incoming HBV nucleocapsids, hence, impairing the delivery of rcDNA into the nucleus for cccDNA synthesis [50,51,52].

Considering the broad spectrum of functions targeted by CAMs, these compounds are strong candidate drugs against HBV. However, some questions still remain unanswered. A major point of discussion is the ability of CAMs to activate innate immune detection of HBV through pattern recognition receptors (PRR). Indeed, one possibility is that CAMs-induced destabilization of assembled capsid results in release of the HBV genetic material into the cytoplasm where it is recognized by PRRs. Moreover, no study addresses the question whether CAM I-induced core aggregates acquire new biological properties. In fact, CAMI treatment induces core aggregation and redistribution in the different cellular compartments where they can potentially interact with others host factors and/or develop novel functions (e.g., transcriptional regulatory function).

### 3.3. CAM and HBeAg

The HBV genome also expresses Core-related proteins. Specifically, HBV precore ORF codes for a 25 KDa protein, precore, that shares with Core the full N-terminal assembly domain but contains 29 additional amino acids at its N-terminus. Precore protein is further processed both at its N-terminus and C-terminus resulting in a 17 KDa long protein, the HBeAg that conserve the full assembly domain and a 10-amino acid leader peptide at its N-terminus [4,58]. Although HBeAg possesses an intact assembly domain, it does not form a capsid structure because of the presence of a disulfide bridge between cysteine 7 in the leader peptide and the cysteine 61, resulting in a totally different quaternary structure [17,59]. HBeAg is secreted as a soluble dimer from hepatocytes. Several studies shed light on the role of HBeAg in HBV persistence and chronic infection. Indeed, HBeAg is known to inhibit the innate as well as the adaptative immune response against HBV [60,61,62]. Moreover, HBeAg negatively impacts therapies aiming at restoring an immunological response against HBV. Indeed, higher level of HBeAg are negatively correlated with the treatment response rates to PEG-IFNα [63,64].

Recently, several works reported that treatment with specific CAM molecules also reduces the HBeAg levels in cellular cultures as well as in mice [50,65]. Specifically, CAM molecules inhibit HBeAg secretion by impairing the precore post-translational processing and inducing its nuclear accumulation [65]. Considering the negative impact of HBeAg on PEG-IFNα treatment, one can speculate that CAM treatment may be helpful to restore the efficacy of immunological treatments by impairing the secretion of HBeAg.

## 4. CAM Molecules in Preclinical and Clinical Development

The ultimate objective of chronic HBV treatments is to clear the virus and to reduce liver diseases, such as cirrhosis, and prevent HCC. The ideal HBV therapy should be able to definitively eliminate the cccDNA. Currently, neither antiviral drugs nor immunotherapies have been developed allowing for such a sterilizing cure. Thereby, the scientific community agrees that a functional cure is a more realistic target to achieve.

A functional cure is intended to reduce HBV DNA to undetectable levels and to induce loss of HBsAg, with or without seroconversion, in patients after six months of treatment discontinuation [66]. The only approved therapies, NUCs or Peg-INFα, can efficiently control viral replication with HBV DNA decreasing to undetectable levels. However, these treatments are life long, as their cessation results in viral rebound and does not affect the HBsAg level. Moreover, NUCs-resistant HBV mutants often appear, thereby making this treatment ineffective, while Peg-INFα therapy suffers from severe side effects and has a limited chance of success.

The core protein is relevant for almost every step of the HBV life cycle, hence blocking core functions with CAM is a promising antiviral strategy (Figure 1). In recent years, several CAM molecules enter in a preclinical or clinical phase of study (Table 1).

NVR 3–778 is a CAM molecule belonging to the SBA (sulfamoylbenzamide) class that affects capsid assembly and abrogates pgRNA internalization. Preclinical investigations have shown its antiviral properties. In vitro and in vivo studies demonstrated that NVR 3–778 efficiently reduces HBV DNA and HBV RNA both in the intracellular compartment as well as in secreted particles [67,68]. However, its development was stopped because of a limited efficacy at clinically feasible doses.

ABI-H0731 is a class II CAM inducing the formation of empty particles. Preclinical analyses in HBV-infection cellular models assess its direct antiviral properties as blocking pgRNA internalization and prevention of cccDNA synthesis. [69]. Moreover, ABI-H0731 demonstrates broad bioavailability and Pharmacokinetic (PK) features in animal models [69]. Phase I clinical evaluation demonstrates its antiviral properties both in HBeAg positive and HBeAg negative patients with a maximum HBV DNA decline of 2.9 log_10_ IU/mL and 2.5 log_10_ IU/mL, respectively. In HBeAg positive patients, the mean HBV RNA decline was log_10_ 2.0 copies/mL, whereas in HBeAg negative patients, the decrease reached undetectable levels [70]. Only mild adverse effects were observed with no ALT flares related to the treatment.

RO7049389 is a CAM I family member showing a potent antiviral property in vitro. Interestingly, RO7049389 treatments causes a strong HBV DNA decrease as well as HBsAg and HBeAg loss in murine AAV-HBV-based models [71]. Recently, in a double-blind phase 1 study, RO7049389 was tested in chronic HBV patients and demonstrated potent antiviral activity with a maximum HBV DNA and RNA reduction (3.33 log_10_ IU/mL and 2.77 log_10_ IU/mL, respectively) in patients following the oral administration of 400 mg of the drug twice a day [72]. However, no significant changes in HBsAg were registered whereas viral rebound was observed after the end of treatment. In this study, only mild or moderate adverse events were observed with the most frequent events being ALT and AST increase in chronic HBV patients but not in healthy volunteers [72].

JNJ-56136379, a CAM II molecule, accelerates the rate of Core assembly in vitro thus resulting in the formation of normal nucleocapsids devoid of genetic material. Moreover, higher concentrations of this CAM II molecule induce a decrease in cccDNA levels in human primary hepatocytes probably by blocking the recycling of newly synthesized nucleocapsid [56]. JNJ-56136379 safety and PK profiles were evaluated in healthy volunteers as well as in chronic HBV patients in a phase I clinical study. The related data show that this drug is well tolerated with mild to moderate adverse events (AE) and only few cases of ALT increase were reported [73,74]. Patients receiving JNJ-56136379 treatment show a dose-dependent reduction of HBV DNA and RNA that falls below a low level of quantification (LLOQ) in more than half patients receiving the maximum dose [74]. However, JNJ-56136379 treatment didn’t affect HBsAg and HBeAg, whereas viral rebound was observed after the end of treatment.

GLS4 is a CAM I molecule belonging to the HAP chemofamily. Phase I study reports GLS4 antiviral properties in chronic HBV patients receiving 120 mg or 240 mg doses, cohort A and B, respectively. The mean declines in level of HBV DNA were −1.42, −2.13 log_10_ copies/mL, respectively, whereas the mean declines in pgRNA were −0.75, −1.78 log_10_ copies/mL [75].

Other CAM molecules already in clinical phase development have been discontinued (Table 1) since they are associated with severe adverse events (AE), such as high ALT elevation. However, several new compounds showing potent antiviral activity in preclinical studies entered in the clinical phase investigation.

GLP26, a glyoxamide derivative, is a novel CAM in preclinical development. This CAM molecule induces the formation of tight, normal nucleocapsid structures [76]. In vitro (HepAD38 cells and primary human hepatocytes (PHH)) as well as in vivo (mice models for HBV infection) studies demonstrated that GLP26 exhibits potent antiviral activity against HBV (EC_50_ in the low nanomolar range) [76,77]. Moreover, GLP-26 alters cccDNA levels in cell culture models whereas a decrease in both HBeAg and HBsAg levels was observed both in vitro and in vivo. Therapeutic combination of GLP26 with ETV shows even stronger antiviral effects in humanized mouse models during treatment as well as after the end of the treatment with viral parameters held at low level or still decreasing 12 weeks after treatment discontinuation [76,77]. Toxicity studies assessed the safety of GLP26 treatment alone or in combination with ETV in cardiomyocytes as well as in humanized mice, whereas PK analyses have shown a good bioavailability (>30 fold the EC_50_) in the blood of monkeys when the treatment administered by food gavage.

ALG-001075 is a CAM II that shows potent antiviral activity in preclinical studies [78]. ALG-000184 is an ALG-001075 prodrug that is currently in clinical phase I development. In vitro experiments in HBV-infected primary human hepatocytes demonstrated that ALG-001075 promotes the reduction of HBV DNA as well as HBV RNA, HBsAg and HBeAg (when added at the time of infection) with an EC_50_ in the low nanomolar range [78]. Moreover, ALG-000184, show an efficient PK profile and excellent bioavailability in rats and dogs. In a clinical phase study, I ALG-000184 shown good safety, and PK and antiviral properties in CHB subjects [79].

To conclude, all CAM treatments show potent antiviral properties against HBV in chronic HBV patients, yet several limitations are present. For now, no CAM compound has been able to trigger a HBsAg decline in patients, and treatment discontinuation is associated with viral rebound. However, preclinical data clearly demonstrate that several CAMs can induce HBsAg loss in mice with a humanized liver. It would, therefore, be interesting to test other treatment conditions in an attempt to reproduce in patients the HBsAg loss observed in murine models.

**Table 1:** List of CAMs in clinical or preclinical development with the associated post-treatment viral parameters in patients.

CAMs	Clinical Phase	Post-Treatment Reduction of Viral Parameters *
NVR 3–778 (Novira, Janssen Pharmaceutica)	Discontinued	DNA 1.97 log_10_ IU/mL, RNA 2.09 log_10_ copies/mL
ABI-H0731 (Assembly Bioscience)	Phase IIA	DNA 2.8 log_10_ IU/mL, RNA 2.0 log_10_ copies/mL
RO7049389 (Roche)	Phase II	DNA 3.3 log_10_ IU/mL, RNA 2.77 log_10_ IU/mL
JNJ-56136379 (Janssen)	Phase II	DNA < LLOQ, RNA < LLOQ
AB-506 (Arbutus)	Discontinued	NA
ABI-H2158 (Assembly Bioscience)	Discontinued	NA
ALG-000184 (ALIGOS therapeutics)	Phase I	DNA 3.8 log_10_ IU/mL, RNA 1.9 log_10_ IU/mL
GLS4JHS (Jilin University)	Phase I/II	DNA 2.13 log_10_ IU/mL, RNA 1.78 log_10_ IU/mL
EDP-514 (Enanta)	Phase I	NA
GLP-26 (Emory University) [77]	Preclinical	NA
ABI-H3733 (Assembly Bioscience)	Phase I	NA

NA: data in patients are not yet available; * reported data represent the max reduction obtained.

## 5. Conclusions

Currently no available therapy can efficiently cure HBV chronic infection by eliminating cccDNA from infected hepatocytes. Thus, an alternative goal of the new therapeutic strategies is to achieve sustained loss of HBsAg for at least six months after the end of the treatment. In that regard, CAMs represent a promising class of therapeutic compounds. In CHB patients CAM treatment alone or in combination with other existing antivirals demonstrate potent antiviral activity against HBV DNA and RNA. Moreover, some CAM chemotypes induce a sustained loss of HBsAg after treatment discontinuation in cellular models as well as in mice. However, the therapeutic conditions used in clinical trials do not allow to observe sustained decline of HBsAg after treatment discontinuation in patients. In the future, additional therapeutic conditions must be conducted to assess the potential of CAMs to induce the sustained loss of HBsAg (e.g., in long-term studies). Moreover, as it is now largely suggested, it is likely that combination therapy with the current anti-HBV drugs would be more successful to eventually induce a decline in HBsAg levels and putatively end with an HBV cure. In this context, combination therapy with CAM and entry inhibitors, siRNA, immunomodulators, or therapeutic vaccines aiming at restoring the immune system may be envisaged in the near future to assess the putative synergistic effect of anti-HBV compounds and eliminate this global health threat.

## Data Availability

Not applicable.
